# The YAP1-MAML2 fusion drives tumorigenesis and sustains tumor growth

**DOI:** 10.1016/j.omton.2024.200900

**Published:** 2024-10-28

**Authors:** Wei Ni, Mu Yu, Rongqiang Yang, Jennifer W. Li, Xin Zhou, Ozlem Calbay, Liya Pi, Jianrong Lu, Shuang Huang, Lizi Wu

**Affiliations:** 1 Department of Molecular Genetics and Microbiology, University of Florida, Gainesville, FL 32610, USA; 2 UF Health Cancer Center, University of Florida, Gainesville, FL 32610, USA; 3 UF Institute of Genetics, University of Florida, Gainesville, FL 32610, USA; 4 Department of Medicine, Brown University, Providence, RI 02912, USA; 5 Department of Anatomy and Cell Biology, University of Florida, Gainesville, FL 32610, USA; 6 Department of Pathology and Laboratory Medicine, Tulane University School of Medicine, New Orleans, LA 70112, USA; 7 Department of Biochemistry and Molecular Biology, University of Florida, Gainesville, FL 32610, USA

**Keywords:** YAP1-MAML2 fusion, fusion oncogene, TEAD/YAP1 transcription, oncogenesis, therapeutic target

## Abstract

The Yes1-associated transcriptional regulator-mastermind-like transcriptional co-activator 2 (YAP1-MAML2 [YM]) fusion protein arises from an intrachromosomal inversion and is implicated in various cancers. However, the oncogenic role of the endogenous YM fusion protein remained undefined. In this study, we employed YM-positive ES-2 ovarian cancer cells as a model to explore the roles of the YM fusion in cancer initiation and maintenance. The YM fusion protein localizes to nuclear speckles and contains bifunctional domains: the YAP1 N-terminal domain interacts with transcriptional enhanced associate domain (TEAD) transcription factors, while the MAML2 C-terminal domain activates YAP1/TEAD-driven transcription. YM exhibited transforming activity, as shown by its ability to induce focus formation in immortalized epithelial cells. YM depletion reduced cancer cell proliferation and survival both *in vitro* and in xenograft tumor models. This effect was correlated with a downregulation of YAP1/TEAD-driven genes essential for cellular proliferation and survival, as revealed by transcriptomic analysis. Importantly, YM-positive cancer cells were sensitive to YAP1/TEAD-targeted pharmacologic inhibition. Collectively, these findings establish the YM fusion as a critical driver of oncogenesis and a promising therapeutic target for cancers harboring the YM fusion.

## Graphical abstract

**Figure:**
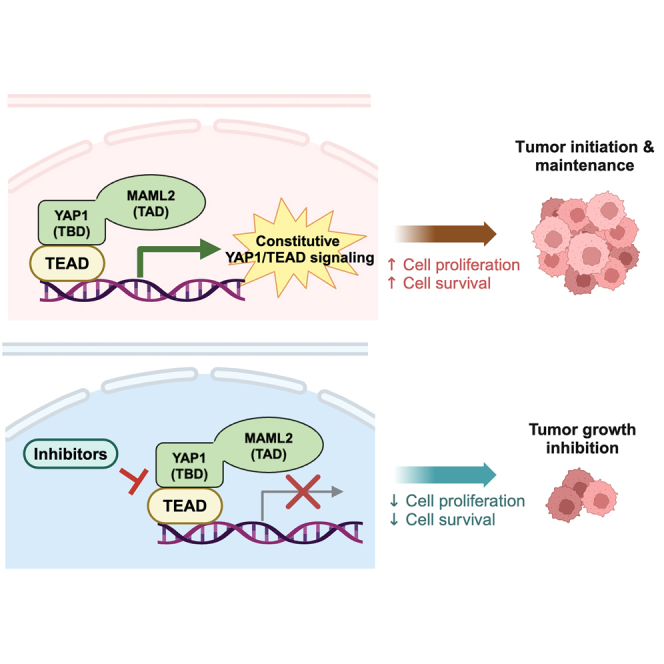

## Teaser

Ni and colleagues show that the YAP1-MAML2 fusion forms nuclear condensates, interacts with TEAD transcription factors, aberrantly activates the YAP1/TEAD-mediated transcriptional program, and exhibits transforming activity. Depletion of the endogenous fusion inhibits the growth of YAP1-MAML2-positive tumors, and cancer cells harboring this fusion are sensitive to YAP1/TEAD inhibitors.

## Introduction

Studies of oncogenic fusion proteins have significantly advanced our understanding of cancer biology by revealing new mechanisms of tumorigenesis and offering opportunities for targeted therapies.^1^^,^^2^ These fusions are potent oncogenic drivers, representing critical targets for both diagnostic and therapeutic interventions. The recurrent Yes-associated transcriptional regulator-mastermind-like transcriptional co-activator 2 (YAP1-MAML2 [YM]) fusion, resulting from intrachromosomal inversion, has been identified in a diverse array of cancers, including nasopharyngeal carcinoma,^3^ ovarian cancer,^4^ skin tumor poroma and porocarcinoma,^5^^,^^6^ thymomas,^7^^,^^8^^,^^9^ parotid gland carcinoma,^10^ and more complex forms of sarcomas^11^ and hemangioendotheliomas.^12^^,^^13^ The identification of the YM fusion in various cancers suggests that it may act as a key driver in activating a common mechanism of tumorigenesis, thereby serving as both a valuable biomarker and a promising therapeutic target.

The YM fusion contains the N-terminal domain of YAP1 ^14^ and the C-terminal domain of MAML2.^15^ YAP1 is a transcriptional co-activator in the Hippo pathway, which controls cell proliferation, apoptosis, and organ size, and is strongly linked to the development and progression of various cancers.^16^^,^^17^ Inactivation of the central mammalian Ste20-like kinase (MST) -large tumor suppressor kinase (LATS) cascade of this pathway leads to the dephosphorylation and translocation of YAP1 to the nucleus, where it partners with the TEAD transcription factors to stimulate gene expression that promotes cell growth and inhibits apoptosis. MAML2 belongs to a family of mastermind-like transcriptional co-activators that are essential for Notch receptor-mediated signaling pathway activation.^18^ Previous studies have shown the transforming activity of the YM fusion in mouse 3T3 cells^6^ and its potential to induce meningioma-like tumors when a truncated form of the fusion (lacking portions of MAML2 sequences equivalent to amino acids (aa) 321–569 or 885–1141 of wild-type MAML2) is introduced into mouse p16-null newborn pup brains.^19^ The YM fusion was linked to reduced cell fitness in MAML2-rearranged cell lines when targeted by guide RNA directed at MAML2 in a CRISPR-Cas9 loss-of-fitness screen.^20^ The overexpression of the YM fusion activates YAP1/TEAD transcription.^6^^,^^19^^,^^21^ However, the specific expression and roles of the endogenous YM fusion are not fully characterized.

In this study, we utilize ovarian cancer ES-2 cells as a YM-positive cancer cell model to investigate the mechanisms and functional roles of the endogenous YM fusion in cancer. We show that the YM fusion forms nuclear condensates, interacts with the TEAD transcription factors, and activates YAP1/TEAD-mediated transcription. The YM fusion sustains the YAP1/TEAD transcriptional program, driving cell transformation and supporting cell proliferation and survival. Collectively, our findings demonstrate that the YM fusion is a potent endogenous oncogenic driver and a promising target for therapeutic intervention.

## Results

### The endogenous YM fusion transcript and protein are expressed in the ES-2 ovarian cancer cell line

**Figure 1:**
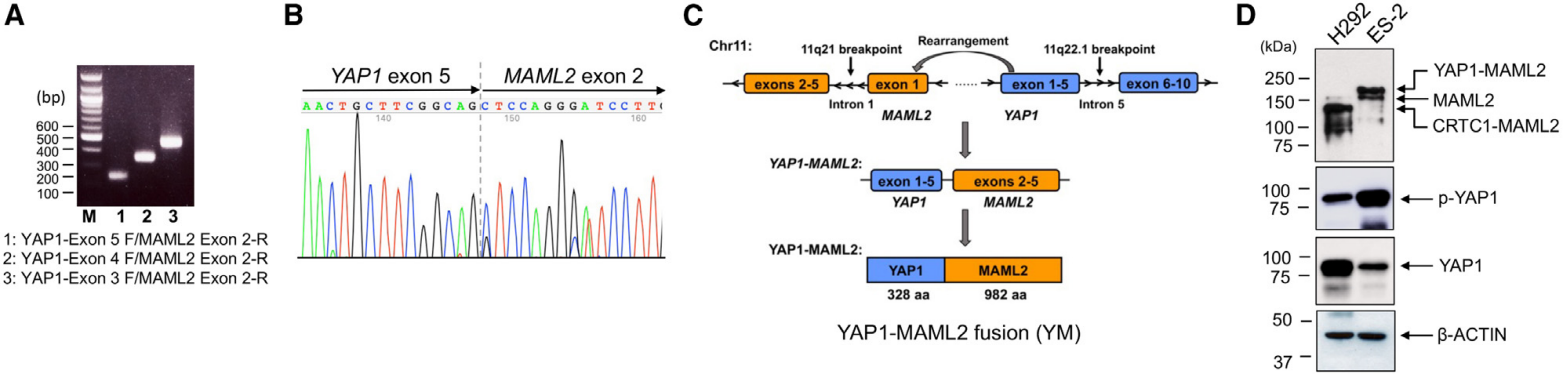
Identification of YM fusion breakpoint and detection of endogenous YM fusion transcript and protein in ES-2 ovarian cancer cell line (A) Amplification of YAP1-MAML2 (YM) fragments using primer sets spanning YAP1 exons 3–5 and MAML2 exon 2. RT-PCR was performed using RNA from ES-2 cells, and amplified products were visualized by agarose gel electrophoresis followed by ethidium bromide staining. (B) Sequence of the fusion junction is shown. (C) Schematic representation of the fusion event, which results in the creation of a novel YM chimeric gene and a fusion protein comprising 328 aa from exons 1–5 of YAP1 (1–328 aa of YAP1) and 982 aa from exons 2–5 of MAML2 (172–1153 aa of MAML2). (D) Detection of endogenous YM protein in ES-2 cells using an anti-MAML2 C-terminal TAD antibody. H292 cells, which express a different fusion protein, CRTC1-MAML2, were used as a control. Note that cells also express native MAML2 proteins.

To characterize the YM fusion junction in ES-2 ovarian cancer cells, we amplified the YM transcript from total RNAs extracted from ES-2 cells using forward primers targeting YAP1 exons 3, 4, and 5, along with a reverse primer for MAML2 exon 2 (Figure 1A). The resulting PCR fragments were subjected to Sanger sequencing, revealing the fusion junction between YAP1 exon 5 and MAML2 exon 2 (Figure 1B). Therefore, this fusion gene results from an intrachromosomal inversion event that fuses YAP1 exons 1–5 at 11q22.1 with MAML2 exons 2–5 at 11q21, generating a chimeric protein spanning 1,311 aa (Figure 1C). Predictably, its encoded fusion protein consists of the N-terminal TEAD-binding domain (TBD) from YAP1 (aa 1–328) and the C-terminal transcriptional activation domain (TAD) from MAML2 (aa 172–1153).

To validate the expression of the endogenous YM protein in ES-2 cells, we performed a western blot analysis using an antibody specific to the MAML2 TAD. As a positive control, lung mucoepidermoid carcinoma H292 cells expressing a different fusion protein, CRTC1-MAML2,^22^ were used. Figure 1D shows that the anti-MAML2 antibody effectively detected both MAML2 (∼160 kDa) and CRTC1-MAML2 fusion (∼130 kDa) proteins in H292 cells. Importantly, this antibody identified MAML2 and YM fusion (∼180 kDa) proteins in ES-2 cells. Moreover, a decrease in normal YAP1 protein levels suggests its expression from an unarranged YAP1 allele in ES-2 cells, while phosphorylation of YAP1 at S397 indicates its phosphorylation by the LATS kinase, rendering it transcriptionally inactive. Therefore, we successfully detected the endogenous YM fusion transcript and protein in ES-2 ovarian cancer cells.

### The YM fusion exhibits nuclear speckles, interacts with TEAD, and activates YAP1-responsive promoter

**Figure 2:**
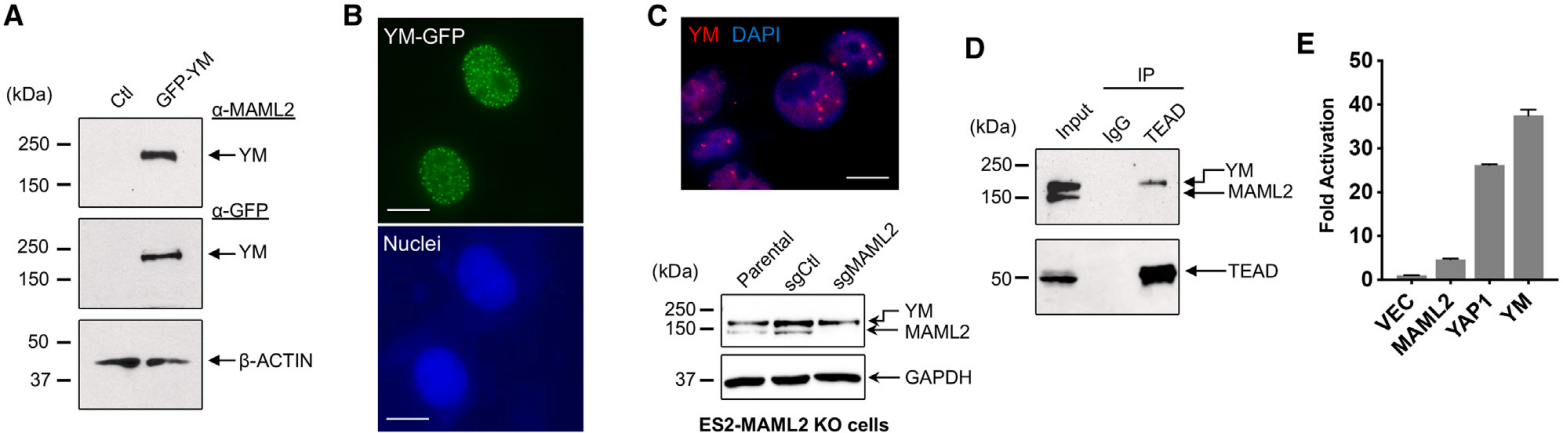
The YM fusion exhibits nuclear speckles, interacts with the TEAD transcription factors, and activates YAP1/TEAD-responsive reporter (A) Expression of the GFP-YM construct was confirmed by transfection of COS7 cells with an empty vector or a vector expressing GFP-YM followed by western blotting using anti-MAML2 TAD and anti-GFP antibodies. (B) GFP-tagged YM exhibited a nuclear speckle pattern (top). The same cells were stained with DAPI to label the nuclei (bottom). Scale bar: 10 μm. (C) Immunofluorescence analysis of ES-2 MAML2 knockout (KO) cells, using anti-MAML2 TAD antibodies, followed by DAPI staining revealed that the endogenous YM fusion protein displays a pattern of nuclear dots (top). The specific MAML2 KO was confirmed via western blot (WB) analysis (bottom). (D) YM-TEAD protein interaction in ES-2 cells was revealed via coIP/WB analysis. Whole-cell protein lysates (750 μg) from parental ES-2 cells were subjected to immunoprecipitation (IP) with anti-TEAD or rabbit IgG (negative control) using Protein A/G beads. IP products were analyzed for TEAD and YM by western blotting. A total of 75 μg whole-cell protein lysate served as an input. (E) The YM fusion was able to activate a YAP-responsive promoter. 293FT cells were plated at 1 × 10^5^ cells/well in 24-well plates overnight and transfected with 10 ng Renilla luciferase control vector, 200 ng YAP/TEAD-responsive firefly reporter vector (8xGTIIC-Luc), and 200 ng of pCMV2 empty vector, pCMV2-MAML2, pCMV2-YAP2, or pCMV2-YM fusion. After 48 h, luciferase assays were performed, and YAP1 promoter firefly luciferase activity was shown as fold activation relative to the basal level with an empty pCMV2 vector (n = 2, mean ± SD).

Because the YM fusion contains LATS phosphorylation sites (S61, S109, S127), we examined its subcellular localization. We constructed a YM fusion consisting of YAP1 (1–328 aa) and MAML2 (172–1153 aa) tagged with GFP at its N terminus and transfected this GFP-tagged YM fusion into COS7 cells. The expression of the GFP-tagged YM fusion was confirmed by western blotting (Figure 2A). We observed strong fluorescence signals predominantly localized in the nucleus as shown by DAPI staining and exhibited speckles (Figure 2B). To assess whether the endogenous YM fusion exhibits such nuclear dot localization, we utilized CRISPR-Cas9 gene editing to target exon 1 of MAML2, achieving selective knockout (KO) of MAML2 while preserving the expression of the YM fusion (Figure 2C, bottom). The resulting MAML2-KO cells were then subjected to immunofluorescence (IF) using anti-MAML2 TAD antibodies to specifically detect the YM fusion protein. The YM fusion protein consistently displayed nuclear speckles, with the number of speckles varying between cells (Figure 2C, top). Consequently, our data indicate that the YM fusion proteins are primarily localized in the nucleus as speckles.

To explore the potential interaction between the endogenous YM and TEAD, we conducted immunoprecipitation (IP) assays using whole-cell lysates from ES-2 cells with immunoglobulin G (IgG; as a negative control) or anti-TEAD antibodies. The immunoprecipitated complexes were subsequently analyzed by western blotting using anti-TEAD and anti-MAML2 antibodies. As shown in Figure 2D, both YM and TEAD were detected in the anti-TEAD immunoprecipitates, demonstrating an endogenous interaction between the YM fusion and TEAD.

Furthermore, to investigate if the YM fusion can constitutively activate YAP1-mediated transcription, we conducted a luciferase reporter assay using a YAP1/TEAD-responsive promoter reporter. YM significantly and robustly activated the promoter reporter, surpassing the activation achieved by MAML2 or YAP1 (Figure 2E), indicating that YM potently stimulates YAP1/TEAD-driven transcription. Overall, these findings suggest that the YM fusion interacts with the TEAD transcription factors in the nucleus, triggering the constitutive activation of YAP1/TEAD signaling independent of external signals.

### The YM fusion exhibits transforming activity in focus formation assays

**Figure 3:**
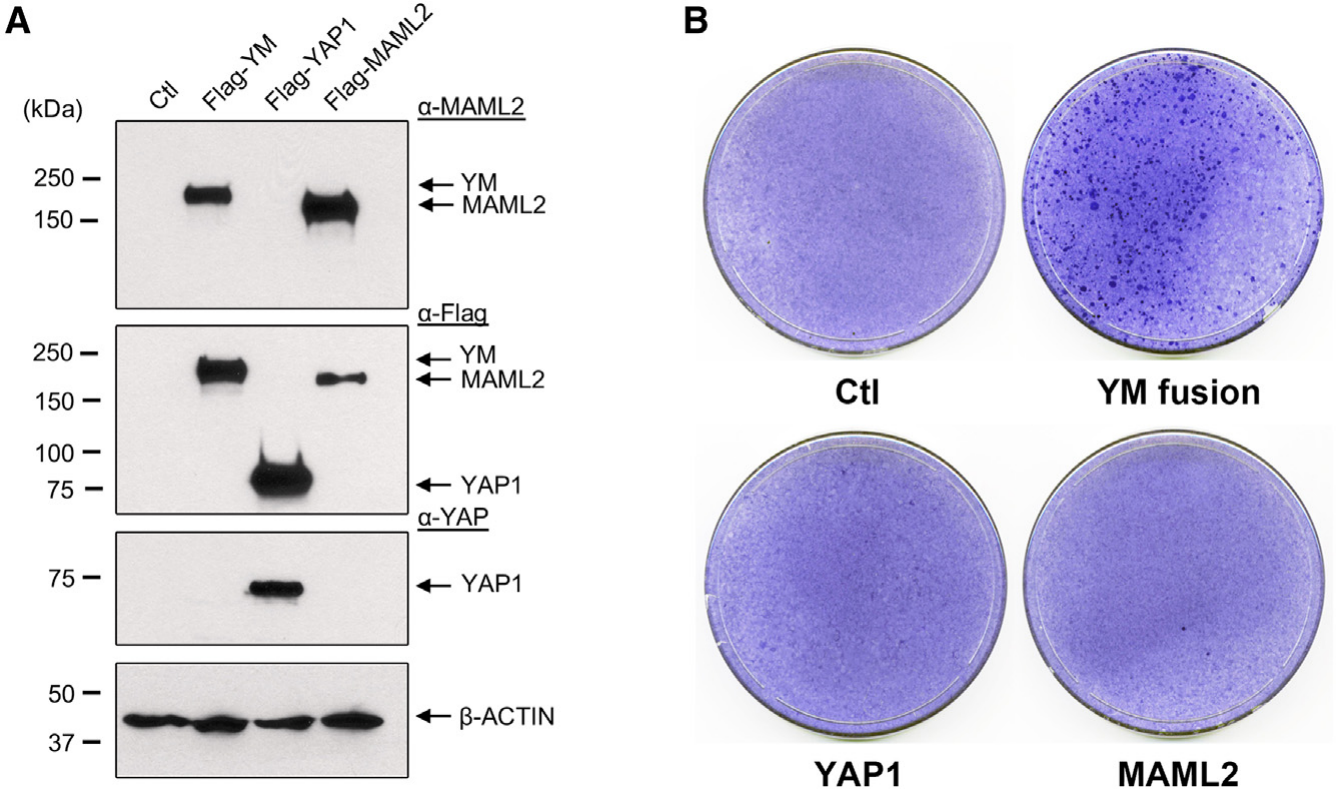
The YM fusion has transforming activity (A) HEK293T cells were transfected with the expression constructs (pCMV2 vector, pCMV2-MAML2, pCMV2-YAP1, and pCMV2-YM), and protein expression was assessed by western blotting using the specified antibodies. (B) RK3E cells were transfected as described above and subsequently evaluated for colony formation at day 15 post-transfection using crystal violet staining.

To evaluate the oncogenic potential of the YM fusion, we performed focus formation assays utilizing E1A-immortalized rat kidney epithelial RK3E cells, a cell model that was previously used to assess the tumorigenic potential of MAML2-rearranged fusions, such as CRTC1-MAML2 fusion.^23^ We transfected RK3E cells with FLAG-tagged constructs of the YM fusion, YAP1, MAML2, and an empty vector control, all of which were validated through western blot analysis (Figure 3A). Focus formation, indicative of cell transformation, was evaluated by staining with crystal violet 15 days post-transfection. The results demonstrated that only the cells expressing the YM fusion, but not those transfected with empty vector, or YAP1 or MAML2, developed foci (Figure 3B). These results indicate that the YM fusion, but not its fusion partners, has a unique oncogenic capacity to transform epithelial cells.

### YM-fusion-positive ovarian cancer cells are dependent on the YM fusion expression for growth and survival

**Figure 4:**
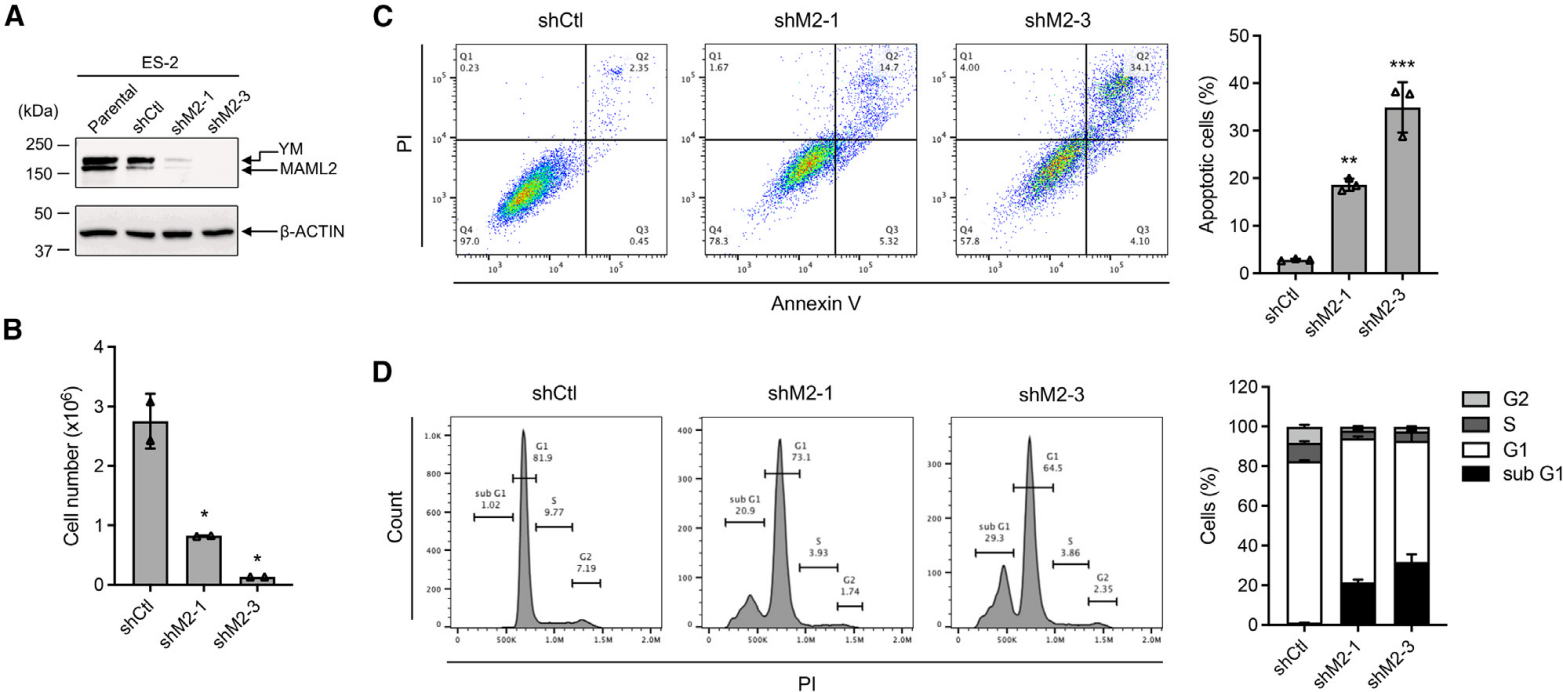
Knockdown of both YM and MAML2 in YM-positive cells reduced cell proliferation, enhanced apoptosis, and promoted sub-G1 cell populations (A) Validation of YM and MAML2 knockdown by western blotting analysis. ES-2 cells were transduced with pLKO.1 lentiviruses carrying shCtl or two shRNAs targeting MAML2 (shM2-1 and shM2-3). Cell lysates were collected 72 h post-transduction, and western blotting was performed using an anti-MAML2 antibody to detect YM and MAML2 proteins. Parental ES-2 cells were included as a control. (B–D) At 72 h post-transduction, ES-2 cells with shCtl, shM2-1, or shM2-3 were harvested for analysis of cell proliferation, apoptosis, and cell cycle. 0.5 × 10^6^ transduced cells were cultured in 10 cm plates for 72 h, and cell numbers were determined by Trypan blue assay (B) (*n* = 2, mean ± SD). Apoptotic cells were assessed via Annexin V/PI staining (C), and cell cycle analysis was conducted using PI staining (D) (*n* = 3, mean ± SD).

To investigate the role of the YM fusion in regulating cancer cell malignancy *in vitro*, we employed short hairpin RNAs (shRNAs; shM2-1 and shM2-3) targeting the 3′ UTR and TAD sequences of the fusion/MAML2, respectively. ES-2 cells were transduced with pLKO.1 lentiviruses carrying scrambled shRNA control (shCtl), shM2-1, or shM2-3. Western blot analysis confirmed that both shM2-1 and shM2-3 reduced the expression of both the endogenous YM fusion and MAML2 (Figure 4A). At 72 h post-transduction, equal numbers of cells from each transduced group were set up to culture for an additional 72 h and were then analyzed for cell proliferation, apoptosis, and cell cycle. Both shM2-1 and shM2-3 reduced cell proliferation (Figure 4B), increased apoptotic cells (Figure 4C), and altered cell cycle distribution, with reduced cells in S phase and increased cells in the sub-G1 phase (Figure 4D). These findings indicate that depleting both YM and MAML2 reduces cell proliferation and enhances apoptosis.

**Figure 5:**
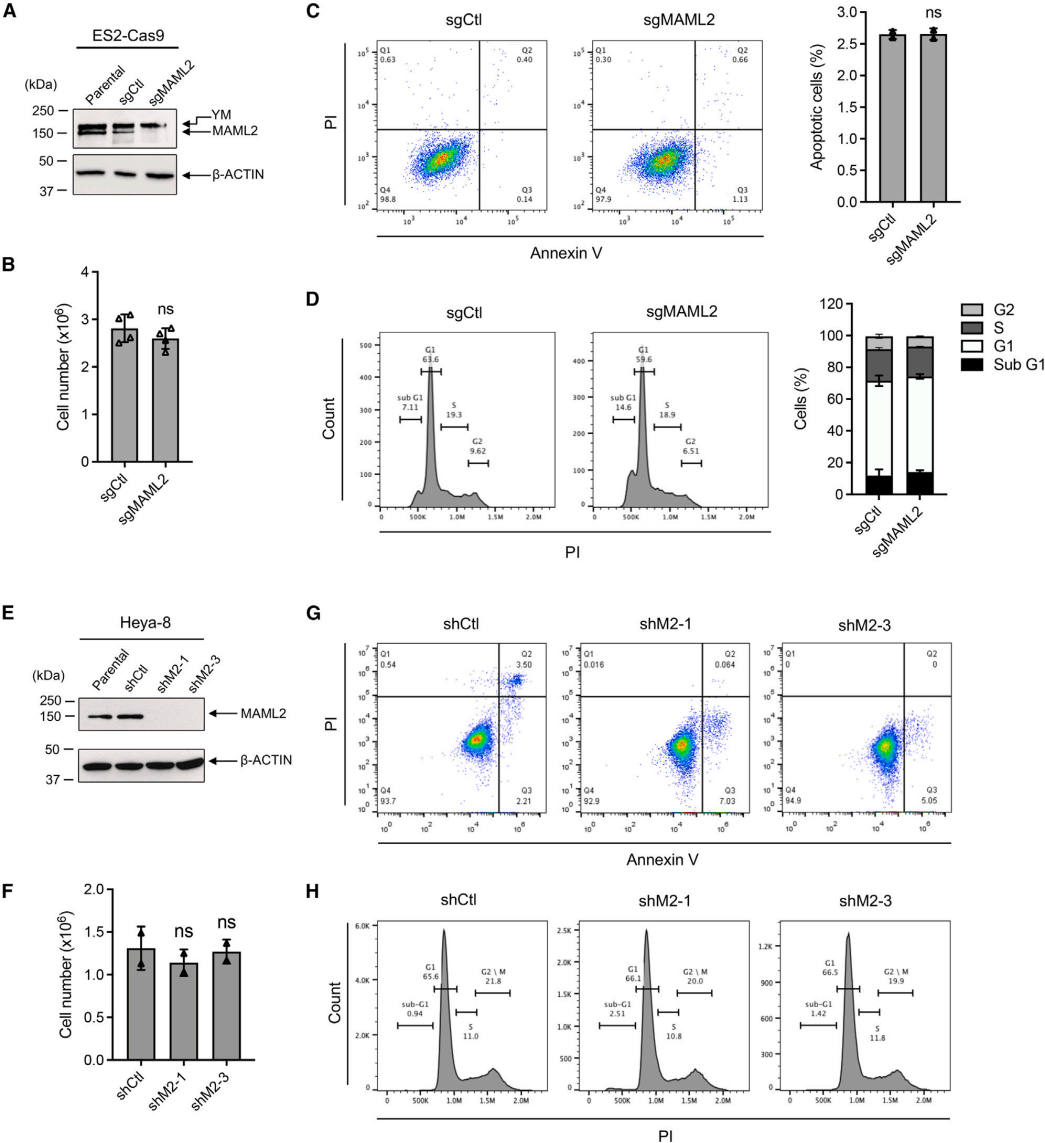
MAML2 knockout in YM-positive and -negative cells had no significant impact on cell proliferation, apoptosis, or cell cycle (A–D) MAML2 knockout in YM-positive ES-2 cells had no effects on cell growth or apoptosis. ES-2-Cas9 cells were transduced with a scrambled sgRNA (sgCtl) or MAML2 sgRNA (sgMAML2) for 72 h. Cells were harvested for western blotting, cell proliferation, apoptosis, and cell cycle analysis. For western blot, MAML2 protein knockout was confirmed using anti-MAML2 antibodies (A). β-Actin served as the loading control. For cell proliferation, apoptosis, and cell cycle, 0.5 × 10^6^ transduced cells were cultured in 10 cm plates for 72 h, and cell numbers were determined by Trypan blue assay (B) (*n* = 4, mean ± SD). Apoptotic cells were assessed through Annexin V/PI staining (C), and cell cycle analysis was performed using PI staining (D) (*n* = 3, mean ± SD). (E–H) MAML2 knockout in YM-negative Heya-8 cells had no effects on cell growth or survival. Heya-8 cells were transduced with pLKO.1 lentiviruses carrying shCtl or two shRNAs targeting MAML2 (shM2-1 and shM2-3). Cell lysates were collected 72 h post-transduction, and western blotting was performed using an anti-MAML2 antibody (E). Parental Heya-8 cells were included as a control. (G and H) At 72 h post-transduction, the transduced cells were also subjected to a similar analysis for cell proliferation, apoptosis, and cell cycle (*n* = 2 per group, mean ± SD).

To dissect the YM-fusion-specific effects, we generated MAML2-KO ES-2 cells using CRISPR-Cas9 with a single guide RNA (sgRNA) directed at exon 1 of MAML2. Western blotting confirmed the near absence of MAML2 protein in MAML2-KO ES-2 cells (Figure 5A). There were no significant differences between MAML2-KO and control cells in terms of viable cell numbers, apoptotic cells, or cell cycle profiles (Figures 5B–5D). Thus, these data suggest that the observed cell phenotypes from YM/MAML2 knockdown (Figure 4B) are primarily attributable to the YM fusion effect. We further confirmed that MAML2 knockdown in a YM-fusion-negative ovarian cancer cell line, Heya-8, did not significantly affect cell growth or apoptosis (Figures 5E–5H), further supporting an essential role of the YM fusion in the sustained growth of YM-positive cancer cells.

**Figure 6:**
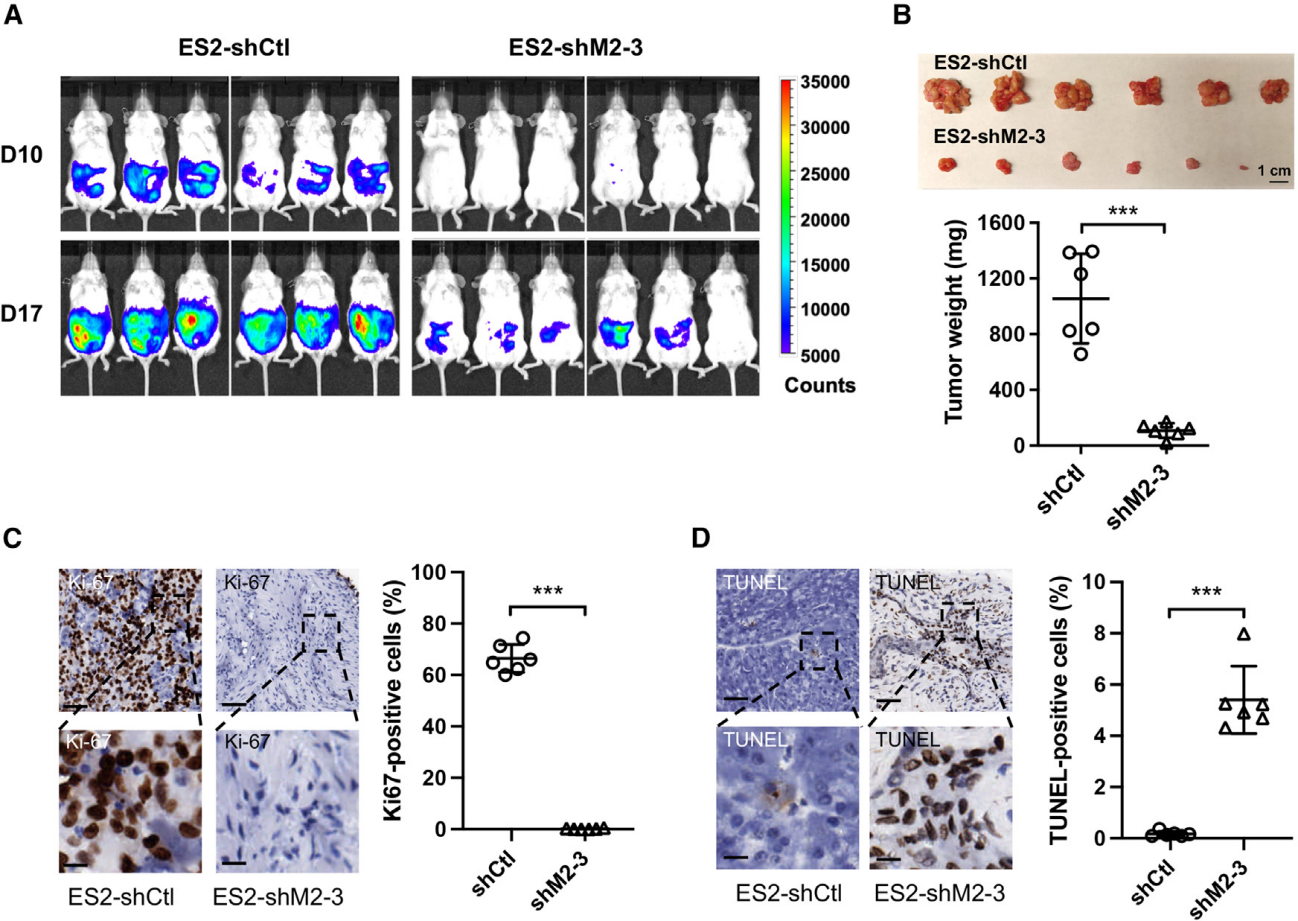
YM knockdown reduced tumor growth Luciferase-expressing ovarian cancer ES-2 cells (ES-2-luc) were infected with pLKO.1 lentiviruses carrying control shRNA (shCtl) and shM2-3 targeting YM/MAML2, respectively. At 72 h post-infection, these two groups of transduced cells (1 × 10^6^ cells/mouse) were intraperitoneally injected into NOD.SCID mice (*n* = 6/group, mean ± SD). (A) Bioluminescent imaging of tumor-bearing mice was performed on day 17 post-tumor cell injection. (B) Images and weights of peritoneal tumor nodules collected on day 17 post-tumor cell injection. (C and D) Representative images of Ki-67 IHC (C) and TUNEL staining (D) of tumors. ImageJ was utilized to analyze and quantify staining-positive cells in sections of individual tumors (*n* = 6 in each group, mean ± SD). Scale bars: 100 μm (top) and 25 μm (bottom).

To assess the effect of YM depletion on tumor growth *in vivo*, we generated ovarian cancer ES-2 cells that express luciferase (ES-2-luc) and subsequently transduced them with pLKO.1 lentiviruses carrying either shCtl or shM2-3 targeting YM/MAML2. Subsequently, these two groups of transduced cells (1 × 10^6^ cells/mouse) were intraperitoneally injected into NOD.SCID mice (*n* = 6/group). Bioluminescence imaging showed a reduction in tumor burden in the shM2-3 group, as indicated by decreased bioluminescence signals on both day 10 and the study’s endpoint, day 17, post-tumor injection (Figure 6A). This reduction was consistent with the decreased weight of peritoneal tumors collected from the shM2-3 group compared to the control group (Figure 6B). Further tumor analysis demonstrated a decrease in cell-proliferative Ki-67-positive cells and an increase in the apoptotic TUNEL-positive cells (Figures 6C and 6D). Overall, these data demonstrate that the YM fusion is critical for sustaining cell growth and survival in YM-positive cancer cells.

### Gene expression profiling analysis revealed YM-fusion-dependent activation of a YAP1/TEAD transcriptional program, including genes critical for cell growth and survival

**Figure 7:**
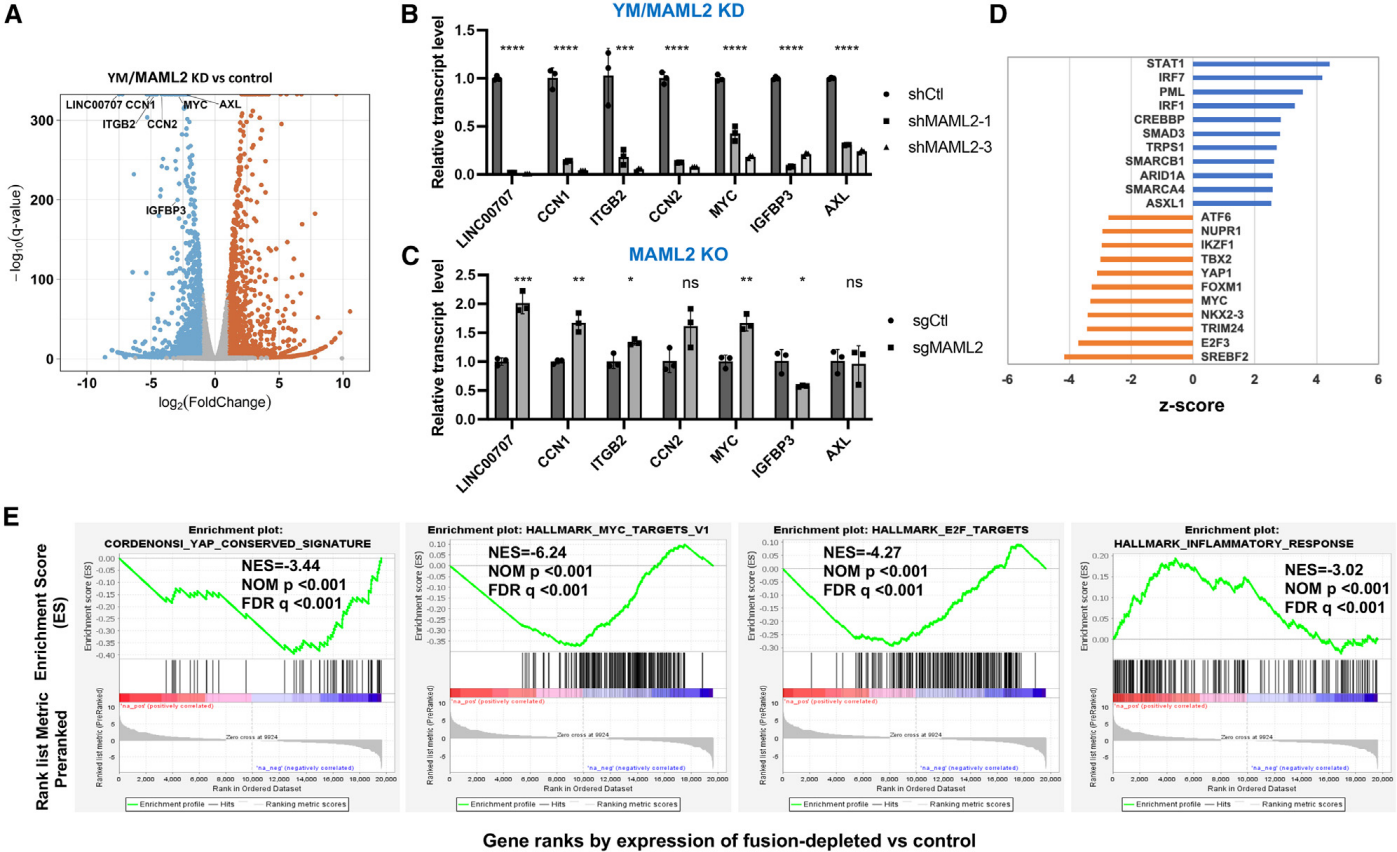
RNA-seq analysis of YM target genes and pathways (A) Differentially expressed genes in ES-2 cells after the depletion of YM/MAML2 are shown in Volcano plot. A few representative YAP1 target genes were indicated. (B and C) The real-time qPCR assays of YM-regulated genes identified from RNA-seq study were conducted using YM/MAML2 knockdown cells (shM2-1 and shM2-3) and the MAML2-KO and their respective control cells (ES-2 shCtl and ES-2 sgCtl) (n = 3, mean ± SD). (D) IPA analysis identified several transcription regulators of the transcriptomic response to YM/MAML2 knockdown. The changes in the activation or inhibition status of transcription regulators were plotted based on their *Z* scores. A positive *Z* score indicates activation, while a negative *Z* score denotes inhibition. (E) GSEA showed that the YAP conserved signature, MYC target, and E2F target gene sets were negatively enriched, whereas the inflammatory response genes were positively enriched in fusion-depleted cells compared to controls.

To elucidate the mechanisms by which YM fusion promotes cancer cell proliferation and survival, we conducted an RNA sequencing (RNA-seq) analysis to compare gene expression between YM/MAML2-depleted (shM2-3) and control (shCtl) ES-2 cells. Differentially expressed genes (DEGs) were identified based on a fold change threshold (≥|2.0|) and a false discovery rate (FDR) cutoff (<0.05), as shown in Table S1. The downregulation of a set of YAP1/TEAD-targeted genes, such as LINC00707, CCN1, ITGB2, CCN2, MYC, IGFBP3, and AXL, was highlighted in the volcano plot (Figure 7A). RT-qPCR analysis confirmed that the downregulation of these genes was specific to the YM/MAML2-knockdown cells, in contrast to MAML2-KO cells (Figures 7B and 7C), indicating that these genes are specifically regulated by the YM fusion.

Ingenuity Pathway Analysis (IPA) revealed the upstream regulators of genes impacted by the depletion of the YM fusion (Figure 7D). Notably, regulators such as SREBF2, E2F3, TRIM24, NKX2-3, MYC, FOXM1, and YAP1 were inhibited, whereas STAT1, IRF7, PML, and IRF1 were activated, as determined by a *Z* score above 3 and a *p* value below 0.05. Corroborating these findings, gene set enrichment analysis (GSEA) demonstrated that the YAP conserved signature, MYC target, and E2F target gene sets were negatively enriched, whereas the inflammatory response genes were positively enriched in fusion-depleted cells compared to controls (Figure 7E). Collectively, these results indicate that the YM fusion not only activates the YAP1-dependent transcriptional program but also contributes to cell growth and survival and potentially modulates the inflammatory response.

### YM-fusion-positive cancer cells are sensitive to YAP1/TEAD inhibition

**Figure 8:**
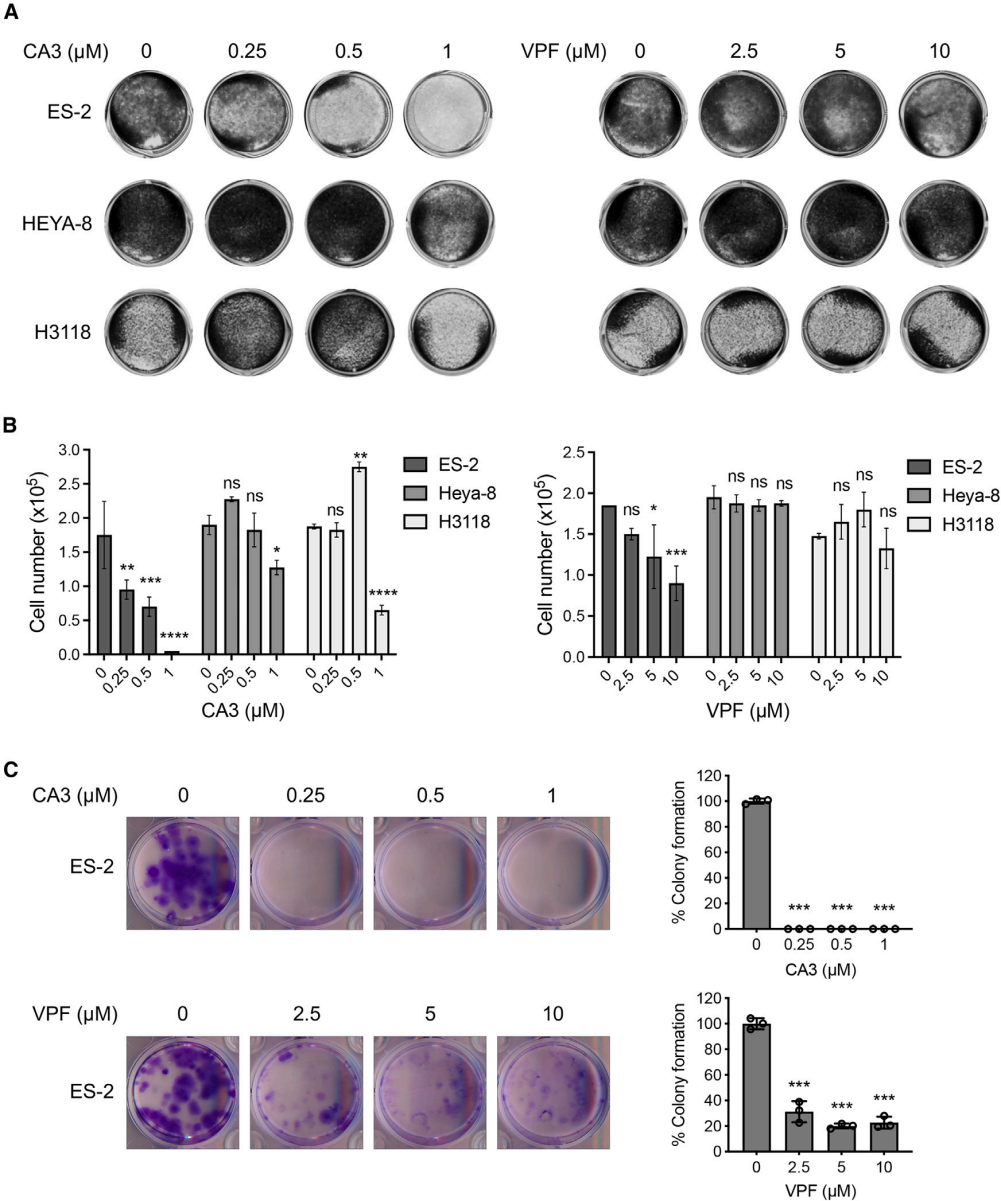
The YM-positive ES-2 cells were sensitive to YAP1 inhibitors (A and B) Cells were cultured in 24-well plates (4 × 10^4^ cells per well for ES-2 and 7.5 × 10^4^ cells per well for Heya-8 and H3118) overnight and then treated with YAP1 inhibitors (CA3 or VPF) at the indicated concentrations for 48 h. Cells were stained using crystal violet (A), and viable cell numbers were determined by Trypan blue assay (B) (*n* = 2, mean ± SD). (C) ES-2 cells were seeded at 500 cells per well in 12-well plates and treated with CA3 or VPF at the indicated concentrations (*n* = 3, mean ± SD). After 14 days of culture, colonies were fixed and stained with crystal violet. The colony areas were quantified using ImageJ and presented as a percentage of the colony area relative to the corresponding DMSO-treated control groups. ∗<0.05, ∗∗<0.005, ∗∗∗,0.0005.

Our data show that fusion-positive ES-2 cancer cells depend on the YM fusion for growth and survival and that the YM fusion drives YAP1-mediated transcription. This dependency suggests that ES-2 cells are susceptible to inhibitors targeting the YAP1/TEAD transcriptional program. Therefore, we treated YM-positive ES-2 cells along with YM-negative Heya-8 cells and mucoepidermoid carcinoma H3118 cells, which carry the CRTC1-MAML2 fusion, with two such inhibitors: verteporfin (VPF) and CA3. VPF is a clinically approved photosensitizer for treating macular degeneration and has been found to disrupt the YAP1-TEAD interaction, thus inhibiting YAP1 signaling.^24^ CA3 was identified through a TEAD reporter transcription screen and was shown to effectively suppress YAP1/TEAD transcriptional activity and tumor growth in mouse models.^25^ The treatment of both inhibitors for 2 days blocked the proliferation of ES-2 cells in a dose-dependent manner, with CA3 showing more potency at lower doses (Figures 8A and 8B). In contrast, there was no or a moderate effect on Heya-8 and H3118 cells. Further, colony formation assays conducted at 14 days post-treatment revealed that ES-2 cells were highly sensitive to both CA3 and VPF (Figure 8C). These findings confirms that YM-positive cancer cells are vulnerable to interventions that target the YAP1/TEAD axis.

## Discussion

The YM fusion has been identified in various cancer types; however, the oncogenic roles and mechanisms of the endogenous YM fusion remain poorly defined. Utilizing YM-positive ES-2 ovarian cancer cells as a model, our study demonstrates that the YM fusion acts as an oncogenic driver by activating the YAP1/TEAD transcriptional program, validating the YM fusion as a therapeutic target.

Previous studies have shown that overexpression of truncated YM fusion proteins induces tumor growth in p16-deficient murine models^19^ and that the sgRNA directed at MAML2 reduced cell proliferation in MAML2-rearranged cell lines.^20^ However, these studies did not fully explore the specific expression and role of the endogenous YM fusion in cancer. Our study addresses this gap by showing the endogenous YM fusion’s expression, its interaction with the TEAD transcription factors, and its essential role in supporting cancer cell proliferation and survival.

The YM fusion in ovarian cancer ES-2 cells comprises two functional domains: YAP1 (1–328 aa) and MAML2 (172–1153 aa). YAP1 (1–328 aa) includes the TEAD-interacting domain that facilitates interaction with TEAD transcription factors,^26^ which was supported by our coIP data showing binding between the endogenous YM fusion and TEAD. MAML2 (172–1153 aa) contributes to robust transcriptional activation through a broad transcriptional domain and directs the YM fusion to the nucleus via a bipartite nuclear localization signal, despite the presence of multiple LATS kinase phosphorylation sites within the YAP1 (1–328 aa) region. Using YM-positive ES-2 cells with KO of endogenous MAML2, which allows specific detection of the YM fusion using antibodies against MAML2 TAD, we showed that endogenous YM exhibits nuclear speckles similar to GFP-tagged YM proteins. This finding aligns with a recent study demonstrating that YM forms nuclear condensates through phase separation.^21^ Disruption of condensate formation impacted target gene expression in engineered 293T cells that express mEGFP-tagged YM, suggesting a role for YM phase separation in promoting gene transcription. Future studies aimed at dissecting the role of YM nuclear condensates in tumor initiation, progression, and maintenance are crucial. Also, YM truncated mutants showed tumorigenic potential,^19^ suggesting that MAML2 321–569 aa or 885–1141 aa are dispensable for tumorigenesis. Further characterization of the functional domains of MAML2 with respect to their ability to form nuclear condensates, transcriptional activation activity, and tumorigenic potential is warranted.

The YM fusion protein is absent in normal cells and arises exclusively in cancer cells due to an intrachromosomal inversion that generates the chimeric YM gene. Using YM-positive ES-2 cancer cells as a model, our knockdown and KO studies established that the YM fusion is critical for sustaining cell growth and survival in YM-positive cancer cells. Transcriptomic analysis further revealed that the YM fusion activates a YAP1/TEAD-mediated transcriptional program essential for cell growth and survival. These findings and those of others^6^^,^^19^^,^^20^^,^^21^ demonstrate that the YM fusion event represents a distinct mechanism for aberrant YAP1 signaling activation that drives tumor development and maintenance. Thus, the fusion emerges as a promising therapeutic target, supported by evidence that YM-positive cells are responsive to pharmacological inhibitors of the YAP1/TEAD transcriptional activity. The potential of targeting this fusion is further highlighted by recent clinical trials of inhibitors that disrupt YAP1-TEAD interactions,^27^ showing promise in selectively inhibiting the oncogenic pathways triggered by the YM fusion.

To our knowledge, this is the first study to characterize the endogenous YM fusion in a patient-derived ES-2 cancer cell line, demonstrating its essential role in supporting cancer cell growth *in vitro* and *in vivo*. It is important to note that this study focuses on a single cancer cell model due to the limited availability of YM-positive cell lines. Two other cancer cell lines, AM-38 (glioblastoma) and SAS (head and neck carcinoma), have previously been associated with the presence of the YM fusion.^20^ Our further analysis using the DepMap portal revealed that the gene effect scores for MAML2 in ES-2, AM-38, and SAS cells were −0.80, −0.53, and −0.58, respectively. These results suggest a dependency on MAML2 and/or the YM fusion in all three cell lines. Since MAML2 is not typically essential for cell growth, the observed growth phenotype is likely driven by the YM fusion. Future research is needed to build on these findings by exploring the therapeutic potential of targeting the YM fusion in various cancer types and assessing the effectiveness of inhibitors that disrupt the YAP-TEAD interaction and transcription.

In conclusion, our study identifies the YM fusion as a critical oncogene with significant potential as both a biomarker and a therapeutic target. We advocate for the inclusion of the YM fusion in cancer screening panels, an essential step for identifying patients with YM-associated cancers. This integration is crucial for guiding future clinical research and developing targeted therapies for patients with YM-positive cancers.

## Materials and methods

### Plasmids and chemicals

A YM fusion cDNA, encoding YAP1 (aa 1–328) and MAML2 (aa 172–1153), was cloned into modified vectors: pFLAG-CMV2 (Sigma) for FLAG-tagged YM fusion, pBIND (Promega) for DB-tagged YM fusion, and pLNCX (Clontech) for GFP-tagged YM expression. Addgene plasmids included YAP/TAZ-responsive luciferase reporter (YAP1/TEAD-luc; 8xGTIIC-luciferase, #34615),^28^ p2xFLAG CMV2-YAP2 (#19045, the correct gene symbol as YAP1),^29^ and pLKO.1-scrambled shRNA vector (#136035) and lentiCas9-Blast (#52962). The lentiGuide-Puro vector, which contains a sgRNA (5′-GATAGCACTGTGCACTCTCG-3′) targeting exon 1 (coding for 1–172 aa) of *MAML2* was previously described.^30^ The lentiviral-based pLKO.1 shRNA constructs shM2-1 (5′-CCCTGTCTAAACTCCAGGATA-3′) and shM2-3 (5′-CCCAAAGCAATTGTTAGCAAA-3′) were previously described.^31^ VPF (T3112) and CA3 (T4309) were purchased from TargetMol.

### Cell lines, transfection, and transduction

H292, ES-2, Heya-8, HEK293FT, and RK3E cells were cultured in Dulbecco’s modified Eagle’s medium (DMEM; Corning) supplemented with 10% fetal bovine serum (FBS; Gibco) and 1% penicillin-streptomycin (PenStrep; Corning). COS7 cells were cultured in RPMI-1640 medium (Corning) with 10% FBS and 1% PenStrep. All cells were grown at 37°C under 5% CO_2_.

Transfections were carried out using Effectene Transfection Reagent (Qiagen) according to the manufacturer’s instructions. Viral production was carried out as described previously.^30^^,^^31^ In brief, HEK293FT cells were transfected with pLKO.1-based or pGuide-sgRNA lentiviral constructs, the packaging plasmid psPAX2, and the envelope plasmid pMD2.G using Effectene Transfection Reagent (Qiagen). Viral supernatants were collected at 48, 72, and 96 h after transfection. Cells were infected over 3 consecutive days by adding viral supernatant to fresh culture medium containing 8 μg/mL polybrene (Sigma) for 6 h.

### RT-PCR

Total RNA extraction was conducted utilizing the RNeasy Mini Kit (Qiagen #74106), followed by reverse transcription into cDNA using the High-Capacity cDNA Reverse Transcription Kit (Applied Biosystems #4368814). Subsequent PCR amplification was carried out using the StepOne Real-Time PCR System with iTaq Universal SYBR Green Supermix (Bio-Rad #1725120). Relative gene expression levels were determined utilizing the comparative ΔΔCt method. GAPDH served as an internal control to normalize gene expression. The following primer sequences were used for LINC00707 (forward, 5′-AAAACTGGAAACCAGCCCCT-3′; reverse, 5′-CACGGTGGCAGTATGGTGAA-3′); CCN1 (forward, 5′-GAAGCGGCTCCCTGTTTTTG-3′; reverse, 5′-CGGGTTTCTTTCACAAGGCG-3′); ITGB2 (forward, 5′-ATCCTGACCCCCAACGACG-3′; reverse, 5′-ATGATCTCGGTGAGTTTCTCGT-3′); CCN2 (forward, 5′-CTCGCGGCTTACCGACTG-3′; reverse, 5′-GGCTCTGCTTCTCTAGCCTG-3′); MYC (forward, 5′-GGAAAACCAGCCTCCCGC-3′; reverse, 5′-CTGCTGCTGCTGGTAGAAGT-3′); IGFBP3 (forward, 5′-GCGCAGCTCCAGGAAATGCTA-3′; reverse, 5′-TGAATGGAGGGGGTGGAACT-3′); AXL (forward, 5′-CAATGGGGACTACTACCGCC-3′; reverse, 5′-GAAGGACCACACATCGCTCT-3′); and GAPDH (forward, 5′-CAATGACCCCTTCATTGACC-3′; reverse, 5′-GACAAGCTTCCCGTTCTCAG-3′).

RT-PCR was also performed to amplify sequences spanning from YAP1 exon 3–5 to MAML2 exon 2 using the following forward primers: YAP1-exon 3-F (5′-GACAACAACATGGCAGGACC-3′), YAP1-exon 4-F (5′-TCCTGATGGATGGGAACAAGC-3′), and YAP1-exon 5-F (5′ACCAGAGAATCAGTCAGAGTGC-3′), along with the reverse primer MAML2-exon 2-R (5′-TTGCTGTTGGCAGGAGATAG-3′). The resulting PCR products were separated on an agarose gel and subsequently purified for DNA sequencing.

### Western blotting and IP

Western blotting and IP were performed as described previously.^32^ The following antibodies were used: anti-GFP rabbit antibody (sc-8334) from Santa Cruz; anti-FLAG (PA1-984B) from Invitrogen; anti-β-Actin (A5316) from Sigma; and anti-MAML2 (4618), anti-YAP1 rabbit mAb (14074), anti-p-YAP1 (Ser397) rabbit mAb (13619), anti-pan-TEAD rabbit mAb (13295) and rabbit (DA1E) mAb IgG XP Isotype Control (3900), and anti-GAPDH (D16H11) XP rabbit mAb (5174) from Cell Signaling Technologies.

### IF staining

ES-2-MAML2-KO cells were grown on coverslips, fixed in 4% paraformaldehyde in PBS for 10 min, and permeabilized with 1% SDS in PBS for 5 min. Subsequently, cells were incubated in blocking buffer (2.5% BSA and 0.1% Triton X-100 in PBS) for 1 h, followed by incubation with anti-MAML2 antibodies (Sigma HPA035223, 1:100 in blocking buffer) for 1 h at room temperature. After washing with 0.2% Triton X-100 in PBS, cells were incubated with goat anti-rabbit IgG H&L Alexa Fluor 568 (Abcam, ab175471, 1:200 in blocking buffer) for 80 min at room temperature in the dark. Following extensive washing, cells were stained with DAPI (Vector Laboratories H-1500) and mounted on slides for imaging. Digital images were captured using a Leica DM6000 B microscope and LAS X software.

### Cell proliferation, apoptosis, and cell cycle

Viable cell numbers were determined using Trypan blue exclusion (Thermo Fisher Scientific). Apoptotic cells were detected using Annexin V/PI staining (BD Biosciences) following the manufacturer’s protocol. For cell cycle analysis, resuspended cells were fixed with 80% cold ethanol overnight, treated with 50 μg/mL RNase, and stained with PI solution. Flow cytometry was performed using a BD Accuri C6 cytometer (BD Biosciences).

### Reporter assay

293FT cells were seeded at 10^5^ cells/well in a 24-well plate and incubated overnight. Co-transfection was performed with 200 ng YAP1/TEAD-luc (8xGTIIC-Luc), 10 ng Renilla luciferase control vector (p-RL), and 200 ng of empty pCMV2 vector, pCMV2-MAML2, pCMV2-YAP1, or pCMV2-YM plasmids. Luciferase assays were conducted at 28 h post-transfection according to the manufacturer’s instructions (Promega, E1910). Firefly luciferase activity was normalized to Renilla luciferase activity for each sample. Reporter activation in each group was reported as relative luciferase activity compared to the empty vector control.

### Colony formation assays

RK3E cells were plated at 10^6^ cells/dish in 10 cm culture dishes and incubated overnight. The cells were then transfected with 2 μg of empty pCMV2 vector, pCMV2-MAML2, pCMV2-YAP1, or pCMV2-YM plasmids. Cells were cultured for 15 days with medium changes twice weekly. Cells were then fixed with 100% ethanol and stained with 0.25% crystal violet in ethanol.

ES-2 cells were seeded at 500 cells per well in 12-well plates (*n* = 3). CA3 (0, 0.25, 0.5, and 1 μM) or VPF (0, 2.5, 5, and 10 μM) was added to the culture 6 h post-seeding. After 14 days of culture, colonies were rinsed with 1× PBS, fixed with 100% ethanol, and stained with 0.25% crystal violet (in ethanol). The stained colonies were scanned, and the colony areas were quantified using ImageJ (v.1.53). Colony formation results were presented as a percentage (%) of the colony area compared to the corresponding DMSO-treated control groups.

### RNA sequencing and data analysis

Two biological replicates of RNA samples were isolated from ES-2 cells transduced with either control shRNA or YM shRNA (shM2-3) lentiviruses for 72 h. Library preparation and sequencing were conducted by Novogene using the NEBNext Ultra RNA Library Prep Kit (Illumina) for non-strand-specific, paired-end sequencing with 150 bp reads on NovaSeq 6000.

Sequencing reads were aligned to the human hg38 reference genome using HISAT2 v.2.0.4 splice-aware aligner,^33^ filtering out ambiguous reads and those with a low mapping quality (MAPQ) score (<10). Transcript quantification was performed using Partek Genomics Suite (v.7.18 Partek, St. Louis, MI) based on hg38 RefSeq transcripts.

Differential expression analysis was conducted using the edgeR package, with significant DEGs defined by read detection in at least one sample (Reads per kilobase per million mapped reads [RPKM] > 1), an absolute fold change ≥2, and an adjusted *p* value ≤ 0.05 using the Benjamini and Hochberg method. Further analysis of DEGs was done using IPA. The GSEA preranked method was performed using the Molecular Signatures Database (MSigDB v.7.1). Gene sets with an FDR q value <0.25 were considered significant.

### Mouse xenograft tumor studies

Two groups of female NOD.SCID mice (JAX NOD.CB17-Prkdc^scid^/J, *n* = 6/group), aged at around 10 weeks, were intraperitoneally injected with ES-2 cells that have been transduced with scrambled shRNA (shCtl) or shM2-3 for 96 h. Each mouse received 1 × 10^6^ cells in 100 μL of a Matrigel (BD Biosciences)/PBS solution (v/v = 1:1). Bioluminescent imaging was performed on days 10 and 17 post-inoculation. On day 17, all mice were euthanized, and the peritoneal tumor nodules developed from each mouse were harvested, photographed, and weighed. Animal studies were carried out following a protocol that was approved by the Institutional Animal Care and Use Committee (IACUC), the University of Florida.

### IHC

Immunohistochemistry (IHC) staining was conducted by the Molecular Pathology Core at the University of Florida following established protocols as previously described.^34^ The positively stained cells were quantified in section scans (visual fields at 5× magnification, 1,000 × 1,000 pixels) of individual tumors (*n* = 6 per group) using ImageJ (v.1.53). The percentage (%) of positively stained cells relative to the total cell count per visual field (*n* = 6) was presented.

### Statistical analyses

Statistical analyses were conducted using GraphPad Prism 10. Specifically, a two-tailed Student’s t test was performed to analyze the differences between two groups, and one-way ANOVA was used for multiple comparisons. Results with a *p* value <0.05 were considered statistically significant.

## Data and code availability

The RNA-seq data were deposited in the Gene Expression Omnibus repository (GEO: GSE267241).

## Acknowledgments

This work was supported by the 10.13039/100000002National Institutes of Health (R01DE023641 and R01CA234351) and the UF Health Cancer Center. We thank the UF Health Cancer Center (UFHCC) Flow Cytometry & Confocal Microscopy Core and NGS Sequencing Core as well as the Molecular Pathology Core at the University of Florida for the technical support.

## Author contributions

W.N. and M.Y. conducted experiments, analyzed data, and wrote the manuscript. R.Y., X.Z., and O.C. carried out *in vitro* experiments. J.W.L. performed data analysis. L.P., J.L., and S.H. provided reagents and contributed to reviewing and editing the manuscript. L.W. designed and directed the study and wrote the manuscript.

## Declaration of interests

The authors declare that they have no known competing financial interests or personal relationships that could have appeared to influence the work reported in this paper.

## Supplemental information

Table S1. A list of differentially expressed protein-coding genes in YM-positive ES-2 cells after depletion of YM/MAML2 ∗Cut-off criteria of absolute fold change of >=2.0 and p value of =<0.05 were used.
